# Assessment of the multidisciplinary education for a major change in clinical practice; a prospective cohort study

**DOI:** 10.1186/1472-6963-9-28

**Published:** 2009-02-11

**Authors:** Ian MR Wright, Chris H Wake, Helene Anderson, Shirley Graham

**Affiliations:** 1 Kaleidoscope Neonatal Intensive Care Unit, John Hunter Children's Hospital, New Lambton Heights, New South Wales, 2310, Australia; 2 Discipline of Paediatrics and Child Health, University of Newcastle, Callaghan, NSW 2310, Australia

## Abstract

**Background:**

New approaches are often introduced to the neonatal intensive care unit (NICU) and other areas of the health service in either a haphazard or cataclysmic fashion. The needs of staff education are often addressed incompletely or too late. Rarely is education assessed after the introduction of a major change. We changed the basis of our NICU respiratory support. We conducted a major educational and support program before this intervention. This study documented and assessed the educational components of this change in our health service provision.

**Methods:**

Senior medical and nursing staff attended training abroad and an education program was applied for one year prior to the change. Multidisciplinary educational support for doctors, nurses and allied health was continued after the change. Assessment was by anonymous questionnaire, prior to change, at one and at nine months. Our hypothesis was that dissatisfaction with education would be greatest at one month.

**Results:**

Both theory education and practical education aspects of the new approach were rated as good to very good and this did not change with time. Difficulty of applying the technique was rated as ambivalent initially but decreased significantly over 9 months until it was rated easy to very easy (p < 0.001). Over all, the change was rated by staff as beneficial, both at the end of the education period and at nine months, with no decrease at one month.

**Conclusion:**

If education and training reaches all staff, with a system of mutual and continued support, even large changes in clinical practice can be achieved without the dissatisfaction with the educational process that is often otherwise seen.

## Background

Neonatal intensive care is an area of medical and nursing care that has been an early convert to evidence-based approaches; the origins of the Cochrane Collaboration are to be found in perinatal medicine [1]. This means that new technologies and innovative approaches are also integral to this still developing field of care. As in other areas of health service, in our extensive experience, over three countries, these new approaches are introduced to the neonatal intensive care unit in a less than ideal way, often in a haphazard and piecemeal fashion. Alternately they may be introduced in a cataclysmic, overnight manner, with no prior education or appropriate support for the medical and nursing staff. Neither approach favours education, understanding or co-operation between staff [2]. The needs of staff education are often addressed after the change has occurred and may be incomplete, leading to neglect of some staff, such as those on night duty [3]. Rarely is there assessment of any educational approaches that are undertaken, even after the introduction of a major change.

Much of the care in neonatal intensive care units through the 1990s had been centred on improving the ventilatory support provided to sick infants using more and more complex technology. After reviewing the evidence and the experience of a number of other centres, we decided to switch to a system of respiratory support that was less invasive and might lead to less iatrogenic damage and thus reduce our incidence of chronic lung disease (CLD) [4-6], a condition with both short term and long term morbidity and mortality for premature infants. We planned to change to a Continuous Positive Airway Pressure (CPAP) based respiratory support, as used in the Columbia Presbyterian Medical Center in New York, as the model for our approach to respiratory care in the neonatal intensive care unit, following a report of its successful implementation away from the immediate support of the Columbia unit [7]. Briefly, this approach centres around the aggressive use of short-prong nasal CPAP, permissive hypercarbia, targeted oxygen saturations and an increased use of blood gas trends, but is more fully described elsewhere [4,7-9]

We conducted an extensive and prolonged educational and support program, prior to, during and after the introduction of this intervention. The purpose of this study was to document and assess the educational components of this change in our service provision.

## Methods

For one year prior to the change, education was conducted. Senior medical staff attended training abroad and then a nurse educator and a clinical nurse specialist visited further units overseas for practical experience. A program of education and assessment of that education was devised, using a variety of modalities suitable for adult learners [10]. Three small-group fixed resource sessions were written covering the theory of Columbia Respiratory Care (CRC) and the practical aspects of the CPAP system to be introduced. Two of these sessions were didactic in nature, based around a series of slides, but with frequent 2-way interaction with the multidisciplinary learners throughout the presentation. The third was a hands-on session allowing a small group to gain practical experience with the CPAP system equipment to be used, in a controlled environment using manikins. These three presentations were repeated over a 3 month period until all day and night staff had had the opportunity to attend each session at least once. With well over 100 staff, many on fractional appointments or on a casual list, this typifies the difficulties facing services such as ours. Many staff chose to attend a session on more than one occasion and this was encouraged. Sessions were attended by nursing staff, medical staff and a number of allied health staff working on the neonatal unit. Practical skills were reinforced by a further visit from external experts that had been involved in original research dealing with this approach to respiratory care of the newborn [8]. Outside learning, with links to further resources provided, was encouraged but not documented. Anecdotally this was taken up by very few. The change to CRC was undertaken on a pre-set date, with the approach to a number of areas basic to neonatal intensive care changing overnight. The clinical aspects involved in our change to CRC have been reported [9] and our outcomes have been assessed by prospective cohort studies [11,12].

After the change had taken place further support was provided by a series of group meetings, attended by both medical and nursing staff, allowing practical solutions to problems to be devised and disseminated through mutual support among the staff. Further information and attention to detail was highlighted by a series of newsletters over the first 6 months, in addition to constant nurse educator support.

We assessed our educational interventions by means of an anonymous questionnaire [see Additional file 1] administered the week prior to the change and then repeated at one month and nine months after the change. Our hypothesis was that any dissatisfaction with education would be greatest at one month. Our sample size was calculated (Statmate, GraphPad Software Inc, San Diego, CA) to demonstrate a 25% decrease in satisfaction with the education received around the time of a major change. The questionnaire asked about the suitability of educational input, both theoretical and practical, about the appropriateness of support for the change, perceived ease of application of the techniques and perceived overall benefit of the new system. Answers were recorded on a 9 centimetre linear scale with annotation to indicate the scale running from very favourable to very adverse, with ambivalence at the mid point. Annotations were transcribed into results in centimetres and analysed as continuous variables (median and interquartile ranges). Data was not normally distributed so was analysed with non-parametric methods, using Instat (GraphPad Software Inc, San Diego, CA).

## Results

Results were available for 62 subjects; repeated analysis was available for 48 members of staff, including senior and junior medical staff and a cross-section of the nursing staff who work within our service. Analysis, using Mann Whitney, of those subjects where repeated measures were available revealed no differences from the pooled analysis, and therefore further analysis was carried out, using Wilcoxon signed rank, on the subset where paired sampling was available. No detailed data were available on those that did not respond to the survey but the proportion of medical to nursing staff was no different and the proportion of part-time to full time staff within the unit was no different between the sampled population and the unit as a whole (p > 0.05).

The education provided in the theory of CRC was rated as good to very good and did not drop below this, even with a statistically significant change at one month (p = 0.018) (Table 1). Although the rating of the training in practical aspects of the new approach was good to very good throughout, there was also a significantly lower rating at one month (p = 0.03). These concerns were lost with time and further experience. Difficulty of applying the technique was rated as ambivalent initially, before its use, but decreased significantly over the 9 month period until it was rated as easy to very easy (p = 0.007). Support was rated as good for the first month and slightly decreased at 9 months to between average and good (p = 0.027). Over all, the change was rated by staff as beneficial, both at the end of the education period and at nine months, with no decrease at one month.

**Table 1:** Questionnaire Results

	**median** ***(IQR)***			**p**		
	**pre**	**1 month**	**9 month**	**Pre-1 month**	**1–9 month**	**Pre-9 month**

Education Theory	1.8 *(0.6–2.7)*	2.1 *(1.4–2.9)*	2 *(1.3–3.0)*	0.018	NS	NS

Education Practical	2 *(1.0–4.8)*	2.8 *(1.9–4.7)*	2 *(1.2–3.2)*	0.03	0.004	NS

Support	1.7 *(0.2–4.0)*	2.3 *(1.5–4.2)*	2.5 *(1.8–3.8)*	0.002	NS	0.027

Ease of application	4.4 *(2.7–4.8)*	3.0 *(2.0–4.7)*	1.3 *(0.4–2.9)*	NS	< 0.001	0.007

Effectiveness	1.7 *(0.1–4.1)*	1.5 *(0.5–2.5)*	1.6 *(0.5–2.1)*	NS	NS	NS

Linear scale scores ranged from 0 – 9, with low scores being beneficial and high scores adverse, with a neutral point at 4.5. Number of subjects = 48. NS = p > 0.05 Wilcoxon signed rank test on the difference between paired samples

## Discussion

In contrast to the more common methods of introduction of change in many healthcare environments, we devised a specific education program intended to inform and empower all members of staff including senior medical staff, junior medical staff, nurse practitioners, permanent and casual nursing staff and our allied health staff. In addition we continued the support and the education process for some months after the change to CRC was introduced.

A number of problems with the introduction of innovation are predictable in the NICU environment, including but not restricted to, a large complement of staff, large proportion of staff with short hours and a large staff complement on night shifts [3]. Our previous experience of the introduction of much lesser changes, in a less organised, way led to our hypothesis that dissatisfaction would be maximal at one month. Protocol and guidelines are a well established part of the neonatal intensive care environment, making sudden change in many areas a potential threat to sometimes long established local norms.

**Figure 1:**
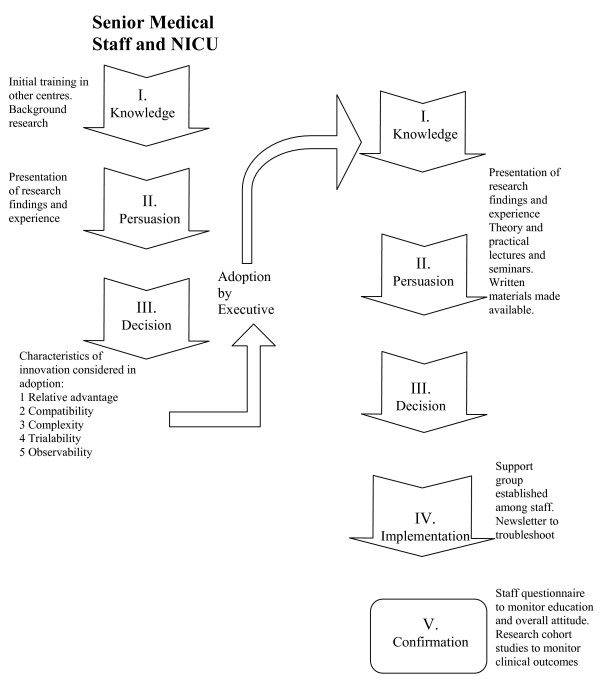
**Schematic of the educational and assessment process for the introduction of Columbia Respiratory Care to our service in the format of Rogers' Model of Diffusion of Innovation**.

Ideally staff would have been randomised to receive the education period or not, to fully establish the effectiveness of the education program. Clearly that would have been very difficult to maintain in our service environment, with a coherent staff body with mixed and varying shift patterns. Although we did not plan it, the intervention that we undertook was essentially that of the Rogers' model of diffusion of innovation (Figure 1) [13]. This was developed to describe the innovation-decision process within large organisations. It was not specifically designed for any particular area, but does provide a useful framework for health service practice changes. In the first stage knowledge was acquired from overseas experience by a few key staff. Next a stage of "persuasion" was undertaken. This in fact occurred in two cycles, as in order to move on to the third stage of "decision" there had to be initial communication of knowledge and persuasion to senior medical and nursing staff in order to achieve a consensus before more widespread education could be undertaken. It could be argued that this second iteration of the "decision" stage was not truly a decision point. We were very clear that the intervention, the introduction of the Columbia Respiratory Care approach, was highly dependent on the involvement and participation of the majority of the staff and therefore a clear rejection at this stage would indeed have led to a failure to proceed to the next stage of implementation. Stage 4, that of implementation occurred at a pre-set time and further support continued at and after this time. Finally, confirmation was both subjective and objective. Subjectively the questionnaire clearly shows that the staff considered the change to be of benefit to the babies cared for within our service. Objectively, our other studies have demonstrated an improvement in outcome for the babies in our care [11,12].

The education prior to a major innovation has been compared to the process of marketing of an idea and has many features in common with commercial marketing. Landrum looked at this process in the context of innovation in a hospital environment; the marketing involved in the "persuasion" component of Roger's theory is clearly applicable to a variety of clinical environments [14]. Our results are compatible with successful "marketing" of the proposed changes, in that the respondents unanimously considered it of benefit prior to the commencement of any change. A study of the introduction of changes in asthma management in Dutch primary health care, using the diffusion of innovation model [15], again showed success and related this partly to the relationship between the demonstration of successful results and continued enthusiasm for the innovations. In our study we also demonstrated both a real improvement and continued perceived effectiveness. The demonstration of improved introduction of innovations has been shown in medical education where immediate feedback of results is provided using live patient simulators [16]. In other healthcare settings innovation has not been associated with sustained change when the perceived benefit has not been apparent [17]. Our use of a graded approach over a long period in part represents resource restriction but also represents the view expressed by innovation and management expert W. Edwards Deming that "Long-term commitment to new learning and new philosophy is required of any management that seeks transformation. The timid and the fainthearted, and the people that expect quick results, are doomed to disappointment" [18].

## Conclusion

In summary we have shown that with a large effort, ensuring that education and training reach all staff and with a system of mutual and continued support, even large changes in clinical practice within a health service can be achieved and sustained. We have shown that this can be done in the neonatal intensive care unit without the dissatisfaction with the process of change that we have so often seen.

## Abbreviations

NICU: neonatal intensive care unit; CLD: chronic lung disease; CPAP: Continuous Positive Airway Pressure; CRC: Columbia Respiratory Care.

## Competing interests

The authors declare that they have no competing interests.

## Authors' contributions

IW conceived and designed the study, undertook the statistical analysis and drafted the paper; CW assisted in study design and reviewed the paper; HA assisted in study design, collected data and reviewed the paper; SG assisted in study design, collected data and helped draft the paper. All authors reviewed and approved the final manuscript.

## Pre-publication history

The pre-publication history for this paper can be accessed here:



## Supplementary Material

Additional file 1 **Questionnaire on Recently Introduced Respiratory Care Method.** The anonymous questionnaire used in the assessment of intervention. Click here for file
