# Supplementary material for: Assessment of the multidisciplinary education for a major change in clinical practice; a prospective cohort study
Source: BMC Health Serv Res. 2009 Feb 11;9:28. doi: 10.1186/1472-6963-9-28 (PMC2645385; doi:10.1186/1472-6963-9-28)
Supplement: Additional file 1 — Questionnaire on Recently Introduced Respiratory Care Method. The anonymous questionnaire used in the assessment of intervention. [file 1472-6963-9-28-S1.doc]

**Questionnaire on Recently Introduced Respiratory Care Method**

Dear Colleagues

As you are aware the neonatal unit recently introduced the Colombia Respiratory Care Method. This questionnaire is designed to look at staff attitudes to Columbia Respiratory Care before introduction, 4-6 weeks, 6 months and 12 months after introduction.

Please mark on the line the position between the two extremes that most represents your opinion. It is a continuous scale, so if you have no feeling one way or the other or if you cannot decide yet etc., then you would mark in the exact centre of the line eg.,

Strongly agree Strongly disagree

X

To take another example, if you felt moderately in agreement then you might mark as below.

Strongly agree Strongly disagree

X

# DO NOT CIRCLE THE WORDS PLEASE MARK ON THE LINE ONLY

1. How many years experience have you had in the care of neonates?

2. Are you a NUM  Neonatologist 

CNS  NNC 

RN 

3. Do you work full time 

part time 

casual 

4. Do you predominantly work:- morning shift 

afternoon shift 

night shift 

1. Which of the following lectures on the newly introduced Columbia Respiratory Care Method did you attend?

Lecture 1  Lecture 2  Lecture 3  Workshop 

2. Do you feel you have had adequate education on the theory of the Columbia Respiratory Care Method?

Very adequate Not at all adequate

3. Do you feel you have had adequate education on the practical aspect of the Columbia Respiratory Care Method?

Very adequate Not at all adequate

4. How do you find the set up of the CPAP circuit?

Very adequate Not at all adequate

5. Do you believe the introduction of the Columbia Respiratory Care Method has made your job?

Very stressful Not at all stressful

6. Do you think staffing levels are adequate at the moment?

Very adequate Not at all adequate

7. Do you think staffing allocations are appropriate?

Very appropriate Not at all adequate

8. Is there adequate support in the nursery for questions regarding Columbia Respiratory Care Method?

Very adequate Not at all adequate

9. Do you believe the use of the Columbia Respiratory Care method will be of benefit to the babies in our unit?

Very beneficial Not at all beneficial

Comments please. Thank you.
